# Supplementary material for: Modeling the potential effects of rooftop solar on household energy burden in the United States
Source: Nat Commun. 2024 Jun 1;15:4676. doi: 10.1038/s41467-024-48967-x (PMC11144185; doi:10.1038/s41467-024-48967-x)
Supplement: Supplementary file 1 — Supplementary information [file 41467_2024_48967_MOESM1_ESM.pdf]

SUPPLEMENTARY INFORMATION

Modeling the potential effects of rooftop solar on household energy burden in the United States

Sydney P. Forrester[1]*, Cristina Crespo Montanes[1], Eric O'Shaughnessy[1], and Galen Barbose[1]

[1] Lawrence Berkeley National Laboratory, 1 Cyclotron Road, Berkeley CA 94720

[*] Corresponding author. Email: SPForrester@lbl.gov

Content of SI

1. Supplementary Figures 1-5
2. Supplementary Tables 1-10
3. Supplementary Methods
4. Supplementary Notes
5. Supplementary References

1. Supplementary Figures

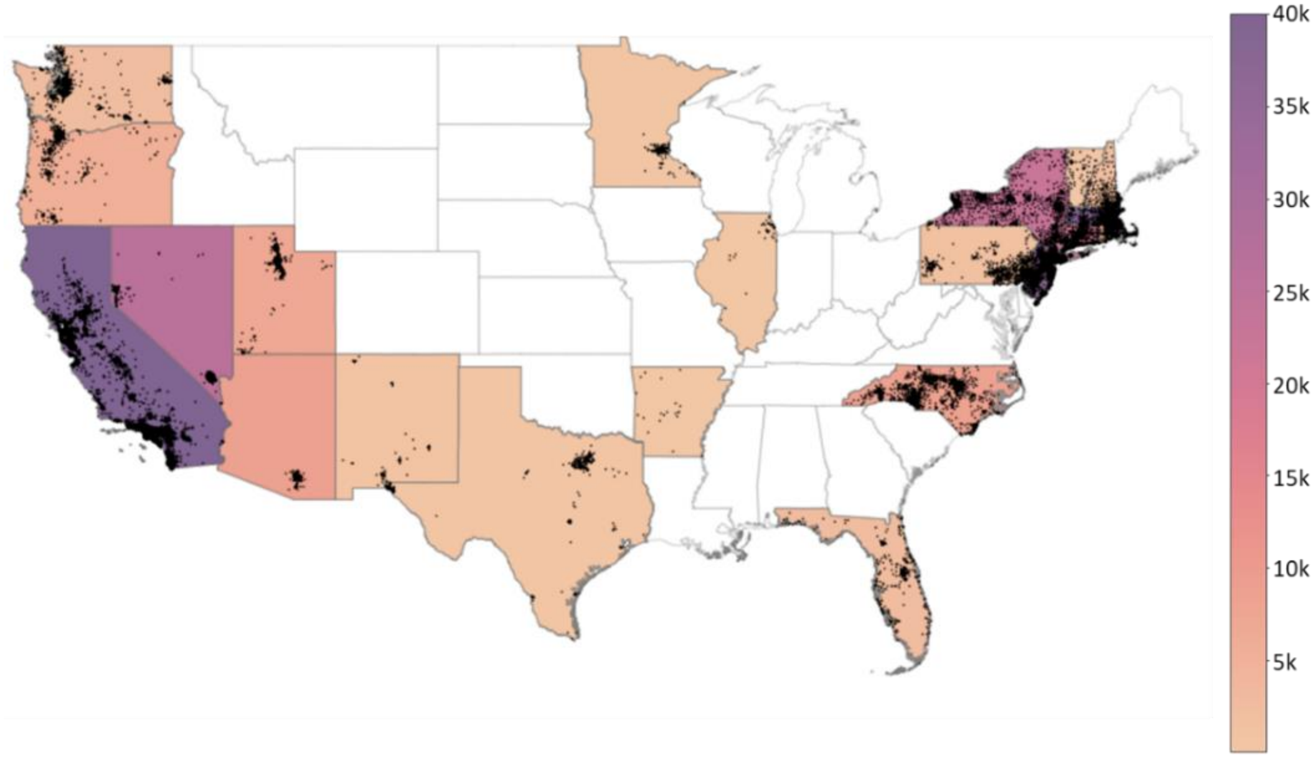

Supplementary Figure 1: Location of the 1M representative sample of adopter households in the study. Darker colored states show higher counts of adopter households.

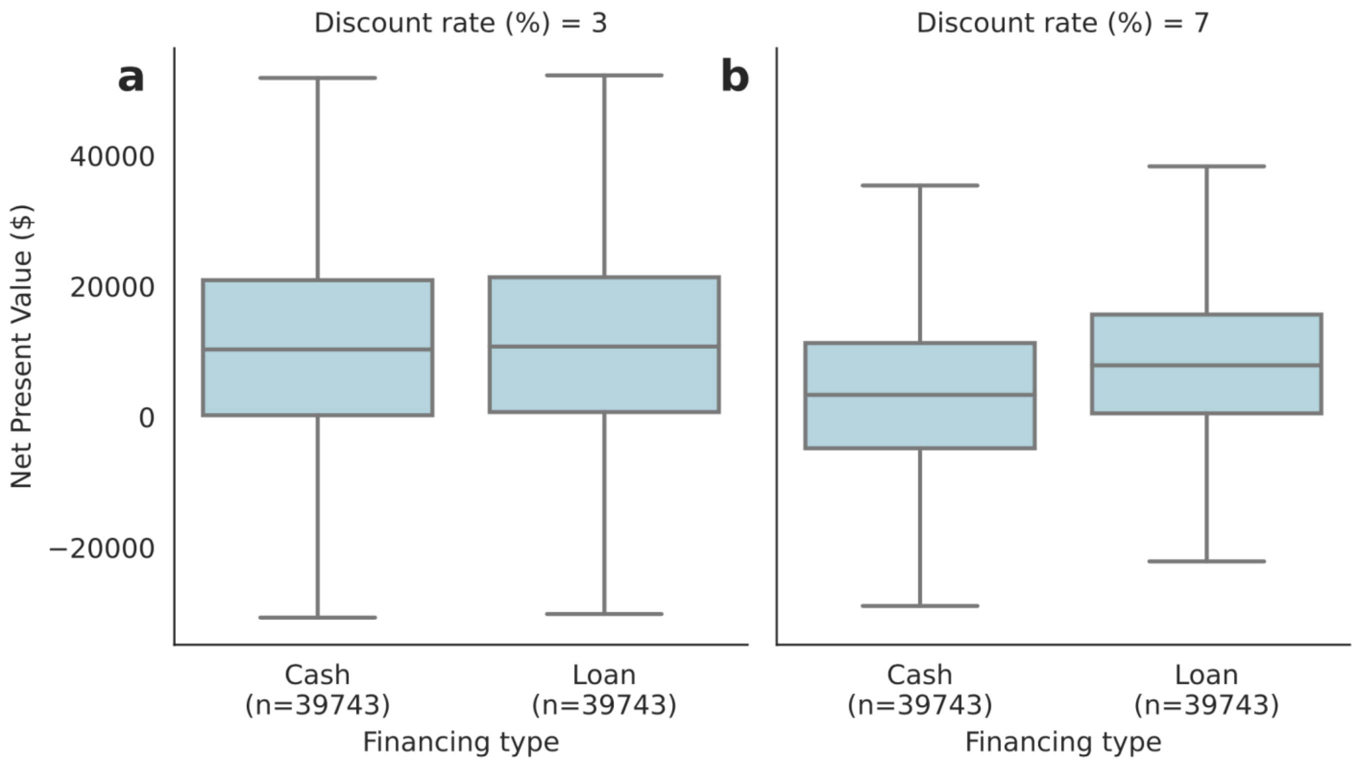

Supplementary Figure 2: Net Present Value of upfront cash vs loan payments for rooftop solar systems installed in 2021. Boxes represent the median and interquartile ranges. Net Present Value distributions, using discount rates of 3% (a) and 7% (b). Whiskers represent minimum and maximum values (excluding outliers). Source data are provided with this paper.

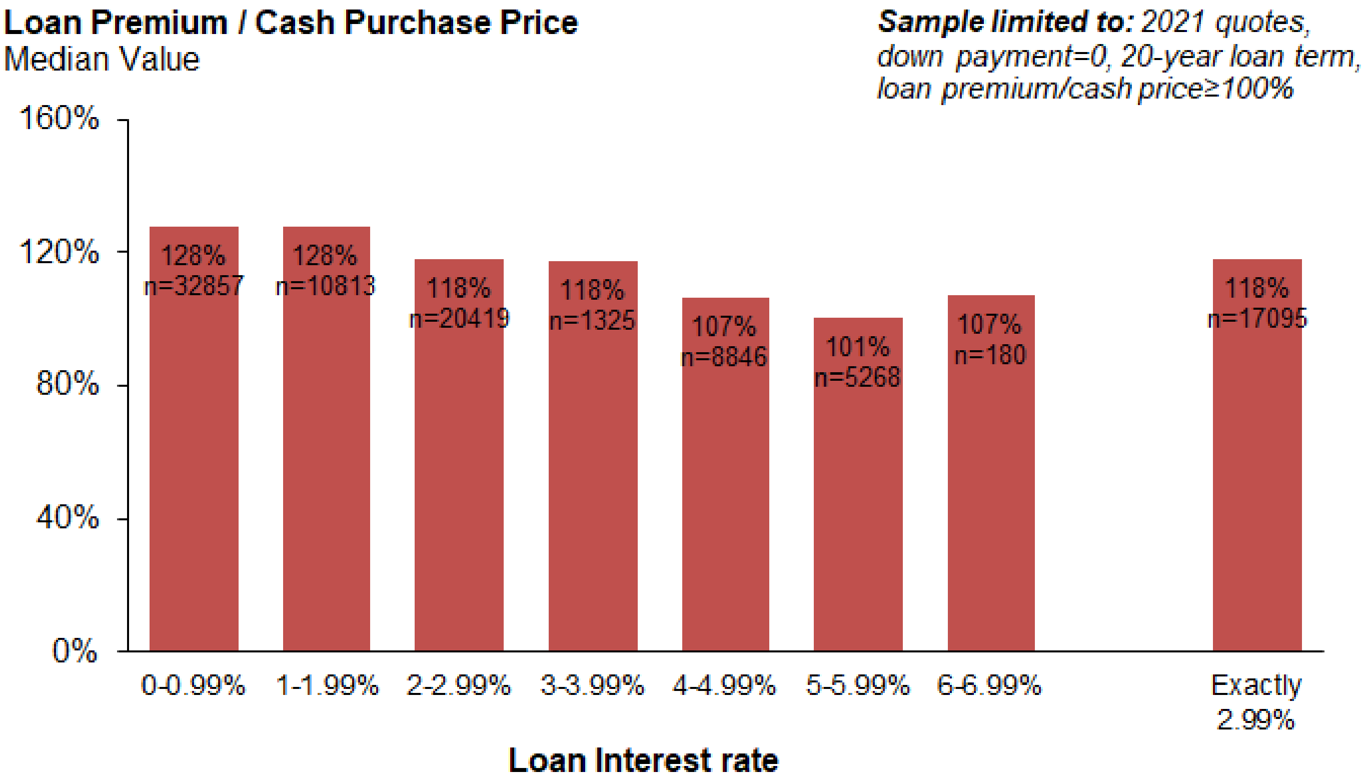

Supplementary Figure 3: Median loan fee calculated from loan amounts and cash-purchase price for EnergySage quotes, for 20-year loans in 2021 with a 2.99% interest rate.

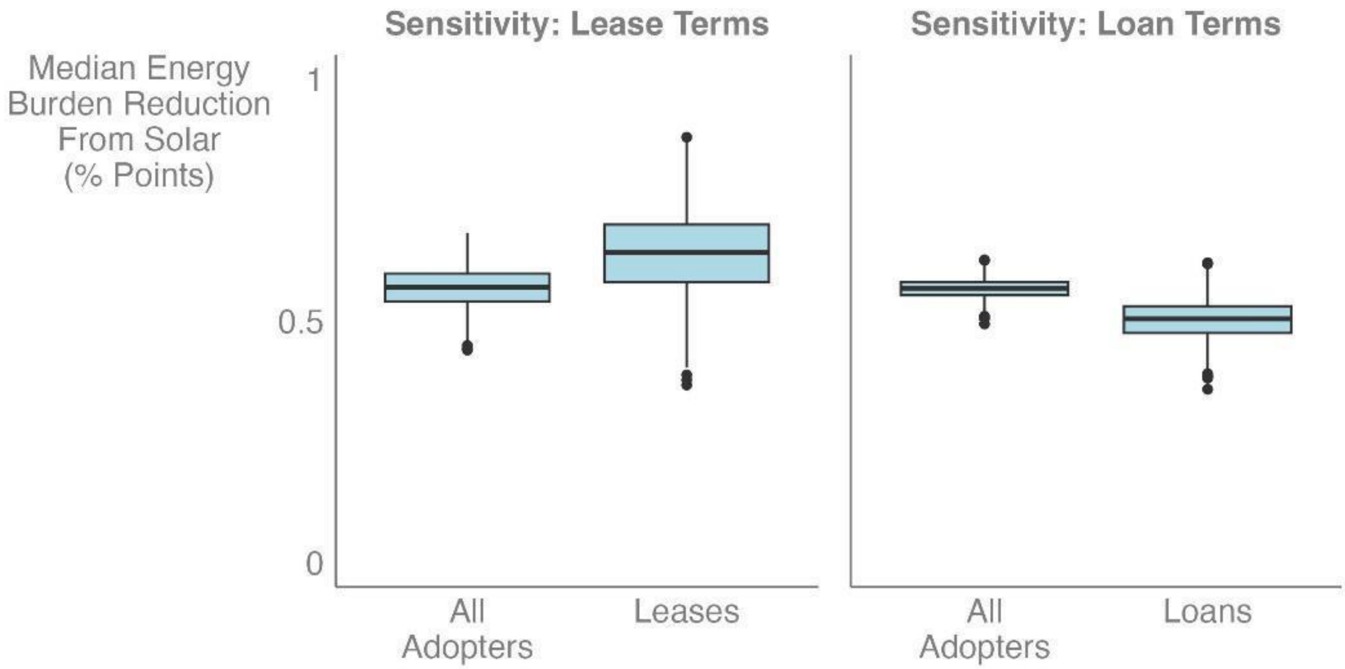

Supplementary Figure 4: Median percentage point change in energy burden reduction across 1,000 iterations of varying lease terms (left) and across 1,000 iterations of varying loan terms (right). Boxes indicate inter-quartile ranges i.e., median, 25th, and 75th percentiles. Error bars depict 5th and 95th percentiles, and points depict outliers. Source data are provided with this paper.

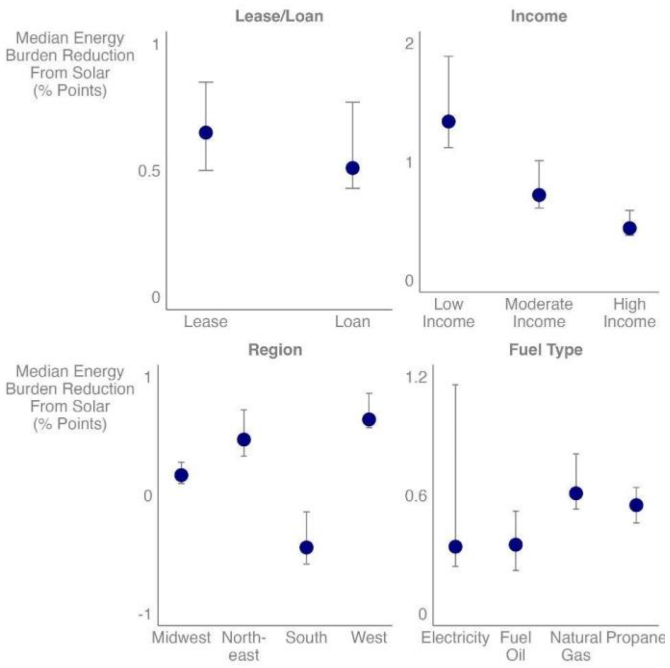

Supplementary Figure 5: Monte Carlo results: Distribution of median energy burden reductions due to solar adoption across the groups analyzed in this paper (ownership structure, income group, region, and heating fuel type). Points represent the average values across all simulations (N=1,000) while the error bars illustrate the 5th and 95th percentile of the distributions over all 1,000 iterations. Source data are provided with this paper.

2. Supplementary Tables

Supplementary Table 1: Summary of data used in this analysis

	Data	Granularity	Description	Source
Step 1	Solar system specs.	Household	System size, street address, cost, incentive levels, installation date, the use of third-party financing, and other system attributes. Estimated income in 2021 at a household level modeled by Experian.	LBNL's Solar Demographics Report[1]
	Property details	Building	Empirical building properties such as square footage, number of stories, heating fuel type, and more	CoreLogic[2]
Step 2	Hourly end-use energy demand	Building model	Closest-neighbor, modeled hourly load profiles determined by matching empirical building characteristics with NREL's ResStock building models	NREL End-Use Load Profiles[3]
	Hourly solar production	County	Hourly kWh production for the centroid of each county, scaled by respective solar system installed capacity	NREL System Advisor Model[4]
Step 3	Electricity tariff	Zip code	Each zip code's most-likely residential tariff pre- and post-solar for 2021, including both fixed and volumetric costs on an hourly basis	Genability[5]
	Heating fuel costs	State	Costs of non-electric heating fuel costs including utility natural gas, propane, and fuel oil, based on state (or region) and month (or year)	U.S. EIA
Step 4	Solar loan and lease terms	State	Typical financial terms developed from aggregated and summarized loan and lease offerings, by state and year.	EnergySage

Supplementary Table 2: Initial number of Tracking the Sun households (HH) in the Solar Demographics report and final number of sampled and matched adopter households in our analysis (counts and percentages).

	**Initial distribution of HH N = 986564**	**Initial distribution of HH (%) N = 986564**	**Final sample distribution n = 500010**	**Final sample distribution (%) n = 500010**
AR	46	0.005	24	0.005
AZ	17495	1.773	8867	1.773
CA	620009	62.845	314227	62.844
CT	34853	3.533	17664	3.533
FL	4906	0.497	2487	0.497
IL	78	0.008	40	0.008
MA	77675	7.873	39367	7.873
MN	682	0.069	346	0.069
NC	16624	1.685	8426	1.685
NH	4006	0.406	2031	0.406
NJ	74209	7.522	37610	7.522
NM	1698	0.172	861	0.172
NV	51356	5.206	26028	5.205
NY	44602	4.521	22605	4.521
OR	10334	1.047	5238	1.048
PA	3122	0.316	1583	0.317
RI	4316	0.437	2188	0.438
TX	835	0.085	424	0.085
UT	14165	1.436	7179	1.436
VT	591	0.060	300	0.060
WA	4962	0.503	2515	0.503

Supplementary Table 3: Variables matched between empirical adopter household sample and EULP modeled buildings

**Variable**	**Variable Type**	**Match type**
State	Categorical	Perfect
Climate zone	Categorical	Perfect
Heating fuel type	Categorical	Perfect
Air conditioning type	Categorical	Perfect
Pool indicator	Binary	Perfect
Number of persons	Numeric	Perfect/ Imperfect
Number of stories	Numeric	Perfect/ Imperfect
Square feet (living space)	Numeric	Perfect/ Imperfect
Year built	Numeric	Perfect/ Imperfect

Supplementary Table 4: Variables matched between empirical adopter household (HH) sample and End Use Load Profile modeled buildings

	Initial distribution of HH (%) N = 986564	Distribution perfect match (%) n' = 606015	Final sample - perfect matches	Final sample - imperfect matches	Final sample distribution (%) n = 500010
AR	0.005	0.003	16	8	0.005
AZ	1.773	1.774	8867	0	1.773
CA	62.845	75.644	314227	0	62.844
CT	3.533	1.982	12011	5653	3.533
FL	0.497	0.566	2487	0	0.497
IL	0.008	0.010	40	0	0.008
MA	7.873	4.768	28897	10470	7.873
MN	0.069	0.058	346	0	0.069
NC	1.685	1.466	8426	0	1.685
NH	0.406	0.053	324	1707	0.406
NJ	7.522	6.252	37610	0	7.522
NM	0.172	0.028	172	689	0.172
NV	5.206	3.914	23721	2307	5.205
NY	4.521	1.682	10191	12414	4.521
OR	1.047	0.388	2353	2885	1.048
PA	0.316	0.172	1041	542	0.317
RI	0.437	0.118	717	1471	0.438
TX	0.085	0.076	424	0	0.085
UT	1.436	0.838	5079	2100	1.436
VT	0.060	0.007	43	257	0.060
WA	0.503	0.200	1212	1303	0.503

Supplementary Table 5: Weighted average annual volumetric and fixed electricity costs across our sample absent solar, compared with EIA data for 2021 by state[9]

	**Volumetric Avg. $/kWh**	**Fixed $/year**	**EIA Volumetric $/kWh - State Avg (for comparison)**
CA	$0.287	$13.040	$0.228
CT	$0.209	$124.744	$0.219
FL	$0.131	$180.251	$0.119
MA	$0.247	$83.939	$0.229
MN	$0.134	$107.757	$0.135
NC	$0.093	$179.497	$0.113
NH	$0.176	$169.782	$0.199
NJ	$0.174	$56.084	$0.164
NM	$0.109	$306.996	$0.135
NV	$0.107	$151.329	$0.115
NY	$0.170	$230.678	$0.195
OR	$0.113	$178.978	$0.114
RI	$0.205	$108.418	$0.223
TX	$0.134	$81.136	$0.121
VT	$0.186	$195.915	$0.193
WA	$0.104	$113.596	$0.101

Supplementary Table 6: Mean and standard deviation of loan interest rates and monthly lease payments per installed capacity, by year. The means are provided as additional information while the standard deviations are the values used to vary (as per a normally distributed set of values) each adopter's deterministic value over the 1,000 iterations, based on year of adoption

	**Interest rate loans (%)**		**Monthly lease payments ($/kW)**	
Year	Mean	Standard Deviation	Mean	Standard Deviation
2013	4.398085	1.667746	11.8045	3.63065
2014	4.953258	1.597751	16.69708	3.602215
2015	5.202252	1.400885	14.95803	4.489026
2016	4.69869	1.335018	15.09683	4.617652
2017	4.459123	1.49145	16.05853	4.631676
2018	4.588306	1.332365	15.71183	3.655411
2019	4.470039	1.129354	17.84004	2.929264
2020	4.028624	1.317391	11.93169	3.607794
2021	2.97979	1.600502	13.43706	4.458521
2022	2.800157	1.628765	13.92194	4.249917
2023	4.493112	1.26838	13.04131	4.167668

Supplementary Table 7: Federal ITC rules applied to PV systems

Interconnection Year	Fed. ITC Level	Max. Incentive Level
2020-2021	26%	N/A
2009-2019	30%	N/A
2006-2008	30%	$2000
Before 2006	N/A	N/A

Supplementary Table 8: State ITC rules applied to PV systems with data from DSIRE database[14]

	State ITC Level	Max. Incentive Level	Installation Year
AZ	25%	$1000	1995-Present
MA	15%	$1000	1979-Present
NY	25%	$5000	1998-Present
NM	10%	$6000	2020-Present
VT	6.24%	N/A	2009-Present
UT	25%	$400	2021
		$800	2020
		$1200	2019
		$1600	2018
		$2000	2001-2017

Supplementary Table 9: SREC levels applied to PV systems at 2021 levels[15]

	2021 SREC [$/kWh]	Notes
DC	$0.416	N/A
MA	$0.283	Install Date 2015+
	$0.331	Install Date 2011-2015
MD	$0.079	N/A
NH	$0.035	N/A
NJ	$0.234	N/A
OH	$0.010	N/A
PA	$0.028	N/A

Supplementary Table 10: Regression results (Y=reduction in EB due to solar), standard errors in parentheses, p-values based on two-sided t-tests in brackets

	(1) Standardized Linear	(2) Log-Linear
EB	0.73*	1.02*
	(0.027) [~0]	(0.01) [~0]
$/kWh	0.49*	1.19*
	(0.005) [~0]	(0.012) [~0]
PV Size (kW)	-0.36*	-0.79*
	(0.004) [~0]	(0.008) [~0]
$/W	-0.35*	-0.9*
	(0.003) [~0]	(0.009) [~0]
Square Footage	0.15*	0.47*
	(0.004) [~0]	(0.01) [~0]
Income	-0.08*	0.04*
	(0.013) [~0]	(0.014) [~0]
Total Annual Bill	0.05*	
	(0.008) [~0]	
Fuel: Oil	-0.81*	-0.8*
	(0.017) [~0]	(0.017) [~0]
Fuel: Gas	-0.37*	-0.32*
	(0.014) [~0]	(0.014) [~0]
Fuel: Propane	-0.24*	-0.19
	(0.14) [0.043]	(0.154) [0.122]
Fuel: Other	0.01	0.12
	(0.129) [0.945]	(0.135) [0.124]
Fuel: None	-0.91*	-0.94*
	(0.052) [~0]	(0.048) [~0]
Northeast	-0.28*	-0.33*
	(0.036) [0.002]	(0.04) [0.001]
South	-0.84*	-0.5*
	(0.039) [~0]	(0.044) [~0]
West	-0.79*	-0.65*
	(0.036) [~0]	(0.04) [~0]
		*p<0.05

3. Supplementary Methods

3.1 Data

A set of adopter households is sampled from LBNL's November 2022 Solar Demographics report[1] with data through 2021 and matched to housing characteristics of modeled buildings from NREL's End-Use Load Profiles (EULP) (see Supplementary Table 1).[3] We set out with a target of modelling 500k solar adopting homes, but aim to keep state-level geographical representativeness of the original approx. 2.5 M solar adopter homes in the Solar Demographics dataset, filtered down to rows for which we have zero missingness for rooftop solar installation size, costs, incentives, date, and income estimates (roughly 1M).[1] Supplementary Table 2 shows the initial and final number of adopter homes per state.

3.2 Matching adopter housing characteristics to modeled buildings

We then find, for each adopter household in our sample, a similar modeled building in EULP. We will then associate the physics-based calculated hourly energy consumption profiles per fuel of EULP buildings to their matched adopter household in our empirical dataset.

First, we filter the EULP models to occupied, single family homes, in order to match the characteristics of our sample. We select only homes with no solar installed for these modeled buildings, since we want to retrieve pre-solar installation consumption profiles for our sample.

Second, we look for variables that we have for both the EULP sample and our adopter sample (from LBNL's Solar Demographic report and CoreLogic's housing stock data). Where necessary, we re-code and re-categorize the values that these variables can take to have equivalent bins and ranges. At this point, it is possible to merge across these nine variables (see Supplementary Table 3) for each individual adopter household to all the EULP building models that are equivalent across these 9 characteristics ("EULP9" models). If there is more than one building model that is matched across these 9 variables to one solar adopter household in our sample, we sample at random from these matched EULP9 models for each adopter household, such that we end up with one-to-one matches. We call this process "perfect matching". For heating fuel type and air conditioning type, we imputed missing values. For heating fuel type, we assumed probabilities from the American Community Survey's 5-year 2017-2021 Table B25040 at the block group level.[6] For air conditioning type, we used the Residential Energy Consumption Survey (RECS).[7]

For air conditioning type, we used RECS to calculate probabilities across four categories (None, Central, Room, Heat Pump) based on state and income group. This involved grouping the RECS microdata by state and income (aggregated into two groups over and under $100,000 annual income) and assessing the relative standard errors (RSE) in R as per RECS guidance.[7] Since air conditioner presence and type are dependent both on location (climate) and income,[8] it was important to generate estimates on both of those indicators while keeping RSE as low as possible, so we analyzed RSE for variations of income groupings (from the available 16 income groups to 2) and spatial groupings (from four regions to every state). Higher aggregation and lower granularity yields lower RSE. Increasing granularity to state level did not produce a high number of large RSE values, however, income groupings introduced some large values across specific AC categories. Nevertheless, RSE remained at lower levels when income was aggregated into two groups (over and under $100,000), which led to our final selection. With these

groupings by state and two income groups, the cases where RSE exceeded 30% were only in locations where estimates indicated low prevalence (likely due to limited sample size). Specifically, across each and every single state/income grouping and AC category, the largest estimate where RSE was higher than 30% was 11% prevalence, which is relatively low. In these specific state/income cases, other AC categories had sufficiently low RSE, so the overall distribution of AC groups was acceptable. In the end, balancing the tradeoffs of RSE versus grouping granularity allowed us to produce likelihood for each adopter to have no AC, central AC, portable/window AC, or heat pump AC based on their state and income. These probabilities, in turn, informed random selections for each household, which was then used for adopters with missing air conditioning values from CoreLogic.

3.3 Matching adopter housing characteristics to modeled buildings

We find approximately 606k "perfect matches" using the aforementioned strategy. The rest of adopter households have not found an EULP building model with shared values in the 9 variables in Supplementary Table 3. We want to ensure spatial representativeness of the original distribution at the state level, however, the proportion of adopter households in each state has changed significantly in the sample of homes that are perfectly matched (Supplementary Table 4). To deal with this, we set an objective number of adopter homes for our adopter sample to be 500k, distributed such that the final sample state-level representation is equal to that of the larger set of 1M households. After the perfect matching process, in some states, the number perfect matches are higher than the target number of final adopter households for that state, so we randomly sample for these states their target number of homes per state. In other states, we do not have enough perfectly matched homes to reach the target number of households per state. In these cases, we use an "imperfect matching" protocol, described below.

The third step consists in matching adopter households with EULP buildings "imperfectly". There are approximately 42k adopter households across all states that still need to be matched with EULP buildings (to reach the target 500k geographically representative sample) but that did not find "perfect" matches (EULP9 models) when merged across the 9 variables in Supplementary Table 3. Thus, in order to match households "imperfectly" we first merge households in the affected states (states with non-zero values in the column "Final sample -imperfect matches" in Supplementary Table 4) with the EULP modeled buildings only based on the 5 non-numerical variables in Supplementary Table 3. Each adopter household can now be matched to many EULP building models based on these 5 variables ("EULP5" model). To select the most similar EULP building for each adopter household from these one-to-many matches, we calculate the Manhattan Distance for the 4 numeric variables in Supplementary Table 3, between the variables in the adopter household and each EULP modelled building. We select from the EULP5 models associated with each adopter household the EULP modeled building with the lowest Manhattan distance across the 4 numeric variables.

Supplementary equation 1: Energy burden (EB) calculation

$$Manhattan\ Distance\ (d) = \sum_{i=1}^{n=4} \left| X_{adopter\ h,i} - X_{EULP\ h,i} \right|$$

Where,

n is the total number of numeric matching variables

$X_{adopter\ h,i}$ refers to the ith matching variable for the adopter household *h*

X_{EULP} refers to the ith matching variable from the EULP5 models associated to adopter household *h*

Finally, each adopter household in the 500k sample gets assigned the hourly energy consumption profiles per fuel that belong to their matching EULP building model. To further calibrate these energy consumption loads to better reflect the characteristics of the adopter household, we scale the total annual energy demand by the actual square footage reported in the adopter household sample, over the square footage reported in the EULP building model.

Finally, we calibrate each adopter's load such that the median ratio of annual rooftop solar generation to estimated electric load across each state/year matches empirical levels determined with EnergySage quotes. These ratios vary from 0.58 (Washington in 2017) to 1.06 (California in 2020). Across all states and years, the median ratio is 1.01. We compare these empirical ratios to the median ratios of the uncalibrated sample by state/year combinations. Each state/year is assigned a correction coefficient such that multiplying all adopters' load within that group would lead to the median matching the empirical data. For example, if the empirical ratio was 1.01 [solar kWh/load kWh] but the calculated, uncalibrated median ratios in the same group were 1.05 [solar kWh/load kWh], the correction coefficient would be 1.05/1.01 or 1.4. This value would be multiplied by each adopter's energy load in that group such that taking the medians of the calibrated ratios would be 1.01 and thus match the empirical data. Only load levels are altered by this coefficient and solar levels are unchanged.

3.4 Electricity rates and fuel costs

To calculate energy bills for the adopter households, we downloaded the electricity tariffs that residential customers were most likely to have in 2021 based on their zip code with and without presence of solar, using Genability software.[5] Of the final set of customers, only 38% of tariffs were the same with and without solar while a majority were moved to different rates. Additionally, absent solar, 6.2% of our sample were on time-sensitive rates. However, with solar adoption this increased to 33.2%. Since we took empirical zip code data, it is important to note that the landscape in 2021 was such that many customers were still not defaulted to time-sensitive rates (for example, this happened in 2022 for the majority of residential customers in California investor-owned utility territories). Moreover, tariffs for solar compensation have also changed quite a bit since 2021. In 2021, the overwhelming majority of solar adopters were compensated via net metering, however, in recent years and months states have begun to explore other options, which would impact energy burden. Again, since we used empirical zip code level data, these tariffs are accurate for the study period and for our purposes (providing a snapshot of how solar has impacted EB historically), but would be an area for further exploration, especially in the context of projected or future EB reduction potential. From these empirical data, we were able to get hourly volumetric costs and fixed monthly costs for each customer, based on their zip code absent and with solar. These values are summarized and compared to state volumetric residential retail prices published by the Energy Information Administration in Supplementary Table 5 using rates absent solar for better comparison.[9] The values do not match exactly because in some cases, our households may cluster in one utility territory or another. In addition, EIA does not report fixed costs.

For non-electric energy costs, we used 2021 costs for residential natural gas,[10] heating oil,[11] and propane.[11] To convert units to a kWh-equivalent (used in EULP), we used a conversion of 138,500 BTU to

gallon of fuel oil,[12] 1,039 BTU to one cubic foot of natural gas,[13] and 91,452 BTU to gallon of propane. [12] Finally, BTUs were converted to kWh at a ratio of 3,412 to 1.

3.5 Financing mechanisms for rooftop solar

In our analysis, we model solar systems that can be financed via loans and lease agreements. To select the financial parameters of the loan contracts and lease agreements, we use solar system quote data from EnergySage.

For loans, we used a dataset of roughly 1.5 million solar loan quotes across the US. For each state/year combination, we took the mode of the loan-term per state and median interest rate for loans of that term.

For leases, we started with 5,000 lease quotes across the U.S. Leases were reported as $/month, which we normalized using the system sizes reported in each quote to $/month/kW. Since this sample was small, we enhanced this dataset with over 20,000 power purchase agreement quotes (another financing option for third-party owned systems). We restricted the PPA quotes to those between 5 and 20 cents/kWh. To convert the PPA costs to a lease agreement metric, we converted the PPA costs into an equivalent $/month/kW lease rate based on expected monthly generation. From this set of data (N = 24,256), we took the lease payment per year and state, in $/kW, if there were mover than ten observations per state/year. If there were less than ten quotes, then this number was replaced by the median lease payment for that year, across all states. Moreover, we do not have lease quotes for systems in Vermont, Nevada, and Minnesota where we do have adopter households with third-party owned systems. We drop these adopter households from our sample since we cannot estimate a lease amount for these states.

Our dataset captures whether the system is host owned or third-party owned, but does not show if host owned systems were loan-financed or paid for upfront with cash. For the purpose of the EB analysis, we treat all host-owned systems as loan-financed. Here, we compare the household financial impacts of loans versus upfront cash-purchases in terms of net present value (Supplementary Figure 2). We calculate the NPV for each financing mechanism (cash or loan) for each adopter household with adoption year 2021 that is flagged as having a host-owned solar system (approximately 40k households). We assume that the reported system costs in the LBNL Solar Demographics report are given loan amounts, which include a loan premium. Hence, for cash payments, we consider an 18% reduction in system costs, in accordance with empirical estimates of loan premium amounts from the EnergySage dataset (See Supplementary Figure 3). We assume a 2.99% interest rate and 20-year loan terms, corresponding to the median interest rate and mode loan term for all EnergySage loan quotes in 2021.

In order to quantify and isolate the sensitivity of EB impacts to lease and loan terms, we ran 1,000 iterations allowing the lease assumptions alone to vary and an additional 1,000 iterations allowing the loan assumptions alone to vary. Supplementary Figure 4 indicates that our results are more sensitive to changes in lease terms than for changes in loan terms. For instance, the interquartile range of EB reductions across all adopters spans 5.8 percentage points when allowing lease terms to vary, compared to 2.8 points when allowing loan terms to vary. Each bootstrap iteration randomly draws an interest rate or a monthly lease payment value from normal distributions with a mean of 0 and a standard deviation equivalent to that observed across all loan or lease quotes available for that year (see Supplementary Table 6). This stochastic adjustment is applied to each customer’s respective loan or lease terms.

3.6 Incentives for solar adoption

To account for off-bill financial impacts of adopting solar, we incorporate empirical data on system costs, incentives, and whether the array is host or third-party owned. The upfront cost is easily determined by subtracting empirical dollar incentives from costs, and then additional incentives are considered, where applicable. For example, the federal (and state, where applicable) Investment Tax Credit(s) are added depending on the year and location of adoption in addition to ownership structure (see Supplementary Table 7 and Supplementary Table 8). Additional, ongoing incentives are also considered such as Solar Renewable Energy Credits (SRECs), which are also awarded to the owner (i.e., to the household in the case of a host-owned system) in applicable states and calculated for calendar year 2021 impacts (see Supplementary Table 9).

4. Supplementary Notes

4.1 Linear regression to explore instances where solar increased energy burden

We wanted to better understand any patterns in adopters' net energy savings, especially to identify what may contribute to solar costs outweighing revenue for 2021. To do so, we define the dependent variable as the difference between EB with and without solar. A positive value indicates a percentage point reduction in EB due to solar adoption, while a negative value indicates that solar was *not* economical for the study period. We then implement two specifications of linear regressions to assess statistical associations between changes in EB and other variables:

An ordinary least-squares regression with standardized inputs (i.e., scaled by subtracting each by the mean and dividing by the standard deviation such that 0 indicates the mean and +/-1 indicates one standard deviation above/below the mean), fuel type, and regional fixed effects

A log-linear specification of the same model. Here, the logged variables are more strongly collinear. In order to reduce collinearity, the variable with the least explanatory power in the first model (annual electricity bill) was dropped.

To reiterate, the dependent variable in these models (change in EB) is a modeled result. These regressions are not empirical models with exogenous errors. Still, the regressions help describe an approximate ranking of the importance of different factors in explaining changes in EB in our model. Greater reductions in EB due to solar were linked to (in order of importance based on standardized coefficients in regressions (1) and (2)): high energy burden pre-solar, high volumetric energy costs, smaller solar sizes, cheaper solar per-Watt prices (after incentives), higher square footage, lower incomes, and larger annual bills (Supplementary Table 10). Although the log-linear coefficients are not directly comparable against each other, the ordering of magnitude of those coefficients is roughly consistent with the ranking just described. Across regions, the models suggest that larger EB reductions were more likely in the Midwest and less likely in the South or West, all else equal. Across heating fuels, households utilizing fuel oil or propane were least likely to see large EB reductions in 2021 due to solar adoption (Supplementary Table 10).

4.2 Statistics

To test whether each population across the groups of interest (i.e., ownership model, income, year of adoption, region, and heating fuel type) was statistically different in terms of their energy burden reduction due to solar and to test how robust our results were to changes in inputs, we conducted several statistical tests. To address the former, we conducted ANOVA and t-tests, and to address the latter we conducted a Monte Carlo detailed below. To summarize, we found that the energy burden reduction of each population in their respective groups were statistically different. We also found our results to be robust over changes to lease terms, loan terms, and rooftop solar shading and soiling.

Independent sample t-test (used when comparing two groups) and one way ANOVA tests (used when comparing more than two groups) showed that energy burden reductions were statistically different across groups. Tests were implemented using Python package scipy.stats. On average, solar reduced household EB for systems financed through lease payments ($n=187087$) by roughly 0.2 percentage points more than for systems financed through loans ($n=234579$) ($t=-38.3$, $p<0.0005$, $DF=413415.7$). Solar adoption reduced low-income household EB by roughly 1.3 percentage points more than for high-

income households ($F=15061.9$, $p<0.0005$, $n=421666$, $df1=2$, $df2=421663$). Solar adoption reduced EB for households using natural gas as a primary heating fuel by roughly 0.3 percentage points more than those using fuel oil ($F=157.2$, $p<0.0005$, $n=421666$, $df1=3$, $df2=421662$). Finally, solar adoption reduced EB for households in the West roughly 1.6 percentage points more than for households in the South ($F=2132.1$, $p<0.0005$, $n=421666$, $df1=3$, $df2=421662$).

In addition to those comparative statistics, we ran a series of Monte Carlo simulations allowing key inputs to vary over 1,000 simulations. We did not allow any variation of empirical data (i.e., rooftop solar installation cost, size, incentives; location; property information at the household level or hourly retail tariff empirical at the zip code level) or values calibrated with empirical data (i.e., load curves matched by empirical property information and calibrated by ratio of solar generation to average annual load). Instead, we focused on allowing variation for the data that was either modeled (i.e., solar generation by county centroid) or aggregated at a less granular level (i.e., financial assumptions aggregated from empirical data to respective state/year combinations). In sum, we explore variations in three key parameters: the interest rate for loans, monthly lease payment values, and the soiling losses of rooftop solar systems.

We generate distributions for the financial parameters (interest rate for loans and monthly lease payment values) using values from historic solar quotes available from EnergySage. Each bootstrap iteration includes an interest rate and a monthly lease payment value that is constructed from: 1) the deterministic value described in Supplementary Methods (calculated based on median values for these parameters per year and state in the EnergySage data) and 2) a stochastic adjustment that is randomly drawn normal distributions with a mean of 0 and a standard deviation equivalent to that observed across all solar loan or lease quotes available for that year in the EnergySage dataset, as applicable (see Supplementary Table 6). The result is that each adopter's interest rate or monthly lease payment is selected randomly for each of the 1,000 iterations from a normal distribution where the mean is set by each adopter's deterministic value and the standard deviation depends on the year of adoption and ownership structure.

In order to stochastically adjust the rooftop solar production of our main results, we modify the deterministic potential rooftop solar production for the centroid of the county where each household is located by applying a stochastic soiling loss factor that is sampled randomly from a normal distribution of soiling loss factors from NREL's Photovoltaic Soiling Map with mean 0.988 and standard deviation 0.015.[50] The soiling factor randomly sampled is capped at 1.

We run 1,000 bootstrap iterations and record the distribution of energy burden with and without solar for each adopter. Our primary goal in running those Monte Carlo simulations was to develop confidence intervals based on the 5th and 95th percentile values from the simulations. Comparisons of the outputs of those simulations across groups provides a robustness check on some key findings in the manuscript. Specifically, the Monte Carlo simulations reiterate that EB reductions are significantly stronger among low-income households and that EB reductions vary significantly across regions, as illustrated in Supplementary Figure 5.

5. Supplementary References

1. Forrester, S., Barbose, G., Darghouth, N., O'Shaughnessy, E. & Montañés, C. Solar Demographics Trends and Analysis. https://emp.lbl.gov/projects/solar-demographics-trends-and-analysis/ (2023).
2. CoreLogic. Property Data Solutions. https://www.corelogic.com/data-solutions/property-data-solutions/.
3. NREL. End-Use Load Profiles for the U.S. Building Stock. https://www.nrel.gov/buildings/end-use-load-profiles.html (2022).
4. NREL. System Advisor Model Version 2020.11.29. https://sam.nrel.gov/ (2020).
5. Genability. Genability Signal APIs. https://www.genability.com/signal/.
6. U.S. Census Bureau. American Community Survey Data. https://www.census.gov/programs-surveys/acs/data.html (2022).
7. U.S. EIA. 2020 RECS Survey Data. https://www.eia.gov/consumption/residential/data/2020/ (2022).
8. Davis, L. W. & Gertler, P. J. Contribution of air conditioning adoption to future energy use under global warming. *Proceedings of the National Academy of Sciences* (2015) doi:10.1073/pnas.1423558112.
9. U.S. EIA. Electricity Data Browser. https://www.eia.gov/electricity/data/browser/#/topic/7?agg=0,1&geo=g0bvg4140j3n&endsec=o&linechart=ELEC.PRICE.US-ALL.A&columnchart=ELEC.PRICE.US-ALL.A&map=ELEC.PRICE.US-ALL.A&freq=A&start=2021&end=2022&ctype=linechart&ltype=pin&rtype=s&pin=&rse=0&maptype= (2022).
10. U.S. EIA. Natural Gas Prices. https://www.eia.gov/dnav/ng/ng_pri_sum_a_EPG0_FWA_DMcf_a.htm.
11. U.S. EIA. Weekly Heating Oil and Propane Prices (October-March). https://www.eia.gov/dnav/pet/pet_pri_wfr_a_EPLLPA_PRS_dpgal_w.htm.
12. U.S. EIA. Units and calculators explained: British thermal units (Btu). https://www.eia.gov/energyexplained/units-and-calculators/british-thermal-units.php.
13. U.S. EIA. Units and calculators explained: Energy conversion calculators. https://www.eia.gov/energyexplained/units-and-calculators/energy-conversion-calculators.php.
14. NC Clean Energy Technology Center. Database of State Incentives for Renewables & Efficiency. https://www.dsireusa.org/.
15. Marex-Spectron. U.S. Green Markets Spectrometer. https://www.marex.com/.
