# Supplementary material for: Modeling the potential effects of rooftop solar on household energy burden in the United States
Source: Nat Commun. 2024 Jun 1;15:4676. doi: 10.1038/s41467-024-48967-x (PMC11144185; doi:10.1038/s41467-024-48967-x)
Supplement: Supplementary file 3 — Reporting Summary [file 41467_2024_48967_MOESM3_ESM.pdf]

Corresponding author(s): Sydney P Forrester

Last updated by author(s): Apr 30, 2024

Reporting Summary

Nature Portfolio wishes to improve the reproducibility of the work that we publish. This form provides structure for consistency and transparency in reporting. For further information on Nature Portfolio policies, see our Editorial Policies and the Editorial Policy Checklist.

Statistics

For all statistical analyses, confirm that the following items are present in the figure legend, table legend, main text, or Methods section.

n/a	Confirmed	
☐	☒	The exact sample size (*n*) for each experimental group/condition, given as a discrete number and unit of measurement
☒	☐	A statement on whether measurements were taken from distinct samples or whether the same sample was measured repeatedly
☐	☒	The statistical test(s) used AND whether they are one- or two-sided<br>*Only common tests should be described solely by name; describe more complex techniques in the Methods section.*
☐	☒	A description of all covariates tested
☐	☒	A description of any assumptions or corrections, such as tests of normality and adjustment for multiple comparisons
☐	☒	A full description of the statistical parameters including central tendency (e.g. means) or other basic estimates (e.g. regression coefficient) AND variation (e.g. standard deviation) or associated estimates of uncertainty (e.g. confidence intervals)
☐	☒	For null hypothesis testing, the test statistic (e.g. *F*, *t*, *r*) with confidence intervals, effect sizes, degrees of freedom and *P* value noted<br>*Give P values as exact values whenever suitable.*
☐	☒	For Bayesian analysis, information on the choice of priors and Markov chain Monte Carlo settings
☒	☐	For hierarchical and complex designs, identification of the appropriate level for tests and full reporting of outcomes
☒	☐	Estimates of effect sizes (e.g. Cohen's *d*, Pearson's *r*), indicating how they were calculated

Our web collection on statistics for biologists contains articles on many of the points above.

Software and code

Policy information about availability of computer code

Data collection	Please see "research sample" for list of data sources and see below for corresponding data collection:<br><br>For Genability data (customer rate data): Signal API was used for collection: https://www.genability.com/signal/<br>For System Advisor Model (solar generation data by county centroid): PySAM API was used for collection. Version 2017.9.5<br><br>No code was used for the rest of the data collection
Data analysis	R was used to create figures. See code availability statement citing the code in the public repository: https://doi.org/10.5281/zenodo.11089676

For manuscripts utilizing custom algorithms or software that are central to the research but not yet described in published literature, software must be made available to editors and reviewers. We strongly encourage code deposition in a community repository (e.g. GitHub). See the Nature Portfolio guidelines for submitting code & software for further information.

Data

Policy information about availability of data

All manuscripts must include a data availability statement. This statement should provide the following information, where applicable:

- Accession codes, unique identifiers, or web links for publicly available datasets
- A description of any restrictions on data availability
- For clinical datasets or third party data, please ensure that the statement adheres to our policy

Source data are available in the figshare repository, 10.6084/m9.figshare.25130498. Additional data that support the findings of this study such as aggregated results are available from the corresponding author upon request. The raw household-specific data (e.g., individual property information, household-level hourly electricity residential retail tariff, individual solar installation information, household income estimates, and personal identifiable information) are protected and not available due to data privacy laws as they were obtained under non-disclosure agreements or via publicly-available paid subscriptions.

The data used as inputs came from a variety of sources:
Proprietary and protected by NDA:
(1) Solar Demographics: Empirical solar installation data (with appended address-linked Experian income estimate, available with license). URL: https://emp.lbl.gov/publications/residential-solar-adopter-income-2 (Berkeley Lab, 2023)

Proprietary and available via license:
(2) CoreLogic Property Data Solutions: Empirical, address-level property information. URL: https://www.corelogic.com/data-solutions/property-data-solutions/ (CoreLogic, 2023)
(3) Genability: Empirical, 2021 utility tariffs and hourly charges. URL: https://www.genability.com/signal/ (Genability, 2023)
(4) Solar loan and lease terms: Empirical, financial terms of solar loans and leases with state and year information (EnergySage, 2023)

Public datasets
(5) End Use Load Profiles: Hourly end-use energy demand by fuel type as well as building characteristics for matching. URL: https://www.nrel.gov/buildings/end-use-load-profiles.html (NREL, 2022)
(6) System Advisor Model. Normalized hourly solar production by county centroid. URL: https://sam.nrel.gov/ (NREL, 2020)
(7) Heating fuel costs: State and year-specific costs of natural gas, propane, and fuel oil in 2021. (US EIA, 2022)

Research involving human participants, their data, or biological material

Policy information about studies with human participants or human data. See also policy information about sex, gender (identity/presentation), and sexual orientation and race, ethnicity and racism.

Reporting on sex and gender	This information was not collected. See "Behavioural and social sciences study design."
Reporting on race, ethnicity, or other socially relevant groupings	This information was not collected. See "Behavioural and social sciences study design."
Population characteristics	These data were not collected. See "Behavioural and social sciences study design."
Recruitment	Participants were not recruited. See "Behavioural and social sciences study design."
Ethics oversight	No human participants were recruited and these data were not collected. Analysis relied solely on preexisting and/or public datasets. See "Behavioural and social sciences study design."

Note that full information on the approval of the study protocol must also be provided in the manuscript.

Field-specific reporting

Please select the one below that is the best fit for your research. If you are not sure, read the appropriate sections before making your selection.

☐ Life sciences ☒ Behavioural & social sciences ☐ Ecological, evolutionary & environmental sciences

For a reference copy of the document with all sections, see nature.com/documents/nr-reporting-summary-flat.pdf

Behavioural & social sciences study design

All studies must disclose on these points even when the disclosure is negative.

Study description	The study is quantitative and relies on several datasets (see below) to conduct descriptive and statistical analyses.
Research sample	The study was centered around 0.5M rooftop solar adopters in the U.S. This study relied on linking multiple preexisting datasets (see "Data". The study sample was selected at random to be proportional to the original dataset of over 2 million homes as in LBNL's Tracking the Sun (2022 edition). While the data from Tracking the Sun is fairly comprehensive (covering over 80% of all rooftop solar

	systems installed in the US to date), coverage varies by location with some states boasting near-perfect coverage and others, less (https://emp.lbl.gov/tracking-the-sun). This dataset is representative of that of Tracking the Sun's data, proportional at the state level. Please see Methods for more information on sampling and filtering along with Supplementary Table 2 for a comparison of the final sample distribution to the initial distribution of households in the Tracking the Sun dataset, by state.
Sampling strategy	The study is representative of the distribution of solar adopter homes across the US, at the state level. We first note the distribution of solar adopters across states based on an original dataset with all solar adopter homes through 2021 (as reported in LBNL's Solar Demographics report). Next, we filter down from this dataset, to to keep only homes for which we can match their key building characteristics (provided by CoreLogic) to EULP buildings (see methods for further details). To preserve the relative distribution of solar adopters across states in the original dataset, we then randomly sample from this sample of matched homes, until we get a target sample size of 500k. This sample size is a compromise to ensure that the inter-state distribution of solar adopters is representative of the original dataset, while keeping a sufficiently large sample for the analysis (20% of all adopters through 2021). Please see the Methods section along with Supplementary Tables 2 and 4 for more information on the spatial distribution of the final sample and the match rate across properties, respectively.
Data collection	The study relied on preexisting data. As such, these data were not collected directly from participants. The data collector was blinded to the experimental condition and the study hypothesis due to the fact that these data are collected on an ongoing basis for a report and are not primarily collected for research purposes. As such, the data were collected independently and before this particular research activity took place.
Timing	The study period is for the 2021 calendar year.
Data exclusions	As stated in "sampling strategy" and Supplemental Information 2 and 3, we excluded data that had missing information for rooftop solar installation size, cost, incentive levels, or income estimates. We then maximized the number of adopter properties that we could perfectly match to building models, excluding some at random by state and including some imperfect matches by state such that the distribution of our study sample resembled the distribution of all U.S. solar adopters.
Non-participation	The study relied on preexisting data. As such, there were no direct participants.
Randomization	The study relied on preexisting data. As such, there were no direct participants. The study does not rely on a control group as we rely instead on a modeled counter-factual scenarios (i.e., absense of solar) for the entire group of confirmed rooftop solar adopters.

Reporting for specific materials, systems and methods

We require information from authors about some types of materials, experimental systems and methods used in many studies. Here, indicate whether each material, system or method listed is relevant to your study. If you are not sure if a list item applies to your research, read the appropriate section before selecting a response.

Materials & experimental systems

n/a	Involved in the study	
☒	☐	Antibodies
☒	☐	Eukaryotic cell lines
☒	☐	Palaeontology and archaeology
☒	☐	Animals and other organisms
☒	☐	Clinical data
☒	☐	Dual use research of concern
☒	☐	Plants

Methods

n/a	Involved in the study	
☒	☐	ChIP-seq
☒	☐	Flow cytometry
☒	☐	MRI-based neuroimaging

Plants

Seed stocks	This is not applicable (checked "n/a" above)
Novel plant genotypes	This is not applicable (checked "n/a" above)
Authentication	This is not applicable (checked "n/a" above)
